# Mortality risk among Autistic children and young people: A nationwide birth cohort study

**DOI:** 10.1177/13623613231224015

**Published:** 2024-02-04

**Authors:** Hien Vu, Nicholas Bowden, Sheree Gibb, Richard Audas, Joanne Dacombe, Laurie McLay, Andrew Sporle, Hilary Stace, Barry Taylor, Hiran Thabrew, Reremoana Theodore, Jessica Tupou, Philip J Schluter

**Affiliations:** 1 University of Otago, Dunedin, New Zealand; 2 National Science Challenge, New Zealand; 3 University of Otago, Wellington, New Zealand; 4 Memorial University of Newfoundland and Labrador, Canada; 5 University of Canterbury, New Zealand; 6 INZight Analytics Ltd., New Zealand; 7 University of Auckland, New Zealand; 8 Victoria University of Wellington, New Zealand; 9 University of Queensland, Australia

**Keywords:** adolescents, autism spectrum disorders, health services, medical comorbidity, pre-school children, school-age children, social services

## Abstract

Autism has been associated with increased mortality risk among adult populations, but little is known about the mortality risk among children and young people (0–24 years). We used a 15-year nationwide birth cohort study using linked health and non-health administrative data to estimate the mortality risk among Autistic children and young people in Aotearoa/New Zealand. Time-to-event analysis was used to determine the association between autism and mortality, controlling for sex, age, ethnicity, deprivation and rurality of residence. The participant population included 895,707 children of whom 11,919 (1.4%) were identified as Autistic. Autism was associated with a significantly higher mortality risk (hazard ratio = 2.35; 95% confidence interval = 1.80–3.06) compared to the general population. In addition, using stratified analyses, we found that this risk was significantly higher among females (hazard ratio = 5.40; 95% confidence interval = 3.42–8.52) compared to males (hazard ratio = 1.82; 95% confidence interval = 1.32–2.52). We also determined that among Autistic young people, mortality risk was significantly higher for those with a co-occurring intellectual disability (hazard ratio = 2.02; 95% confidence interval = 1.17–3.46). In this study, autism was associated with higher mortality in children and young people compared to the non-Autistic population. Increased efforts are required to better meet the health needs of this population.

**Lay abstract:**

Existing literature indicates that Autistic people have shorter life expectancy, but little is known about the mortality risk among Autistic children and young people (0–24 years). We used a 15-year nationwide birth cohort study to estimate the mortality risk among Autistic children and young people in Aotearoa/New Zealand. The study included 895,707 children and 11,919 (1.4%) were Autistic. We found that autism was associated with a significantly higher mortality risk compared to the non-Autistic population. In addition, we found that this risk was significantly higher among females compared to males and for those with a co-occurring intellectual disability. Increased efforts are required to better meet the health needs of this population.

## Introduction

Autism spectrum disorder (autism) is clinically defined as a lifelong neurodevelopmental condition characterised by persistent social and communication differences, sensory issues and restricted repetitive patterns of behaviour or interests (American Psychiatric Association, 2013). Contemporary conceptualisations of autism increasingly apply a strengths-based lens to this heterogeneous condition (Altogether Autism, 2019). Autism prevalence among children in the United States is currently estimated to be 2.8% (approximately 1 in 36 children) (Maenner et al., 2023). Since 2000, this rate has increased more than threefold, due to increased awareness, improved methods of identification and widened definitions (Amaral, 2017).

Among adults, autism has been associated with a significantly increased risk of mortality (Bilder et al., 2013; Catalá-López et al., 2022; Shavelle et al., 2001). For example, in a recent meta-analysis comprising 12 studies, all-cause mortality was found to be over twice as high for Autistic people compared to the general population (Catalá-López et al., 2022). However, scant research has focused on mortality risk among Autistic children and young people. In two identified studies, both based on Korean data, time-to-event analyses revealed that Autistic children have approximately 2.5 times increased mortality risk compared to their general population counterparts (Kim et al., 2021; Yoo et al., 2022).

Previous adult studies have demonstrated that increased mortality risk is primarily due to a multitude of co-occurring conditions (Mouridsen et al., 2008; Pickett et al., 2011), including epilepsy, heart disease and intellectual disability (Hwang et al., 2019; Pickett et al., 2011; Trollor et al., 2017). Mental health and neurodevelopmental conditions among Autistic people also increase the risk of intentional (self-harm) and unintentional injuries (drowning) that may cause death (Hirvikoski et al., 2016; Schendel et al., 2016; Shavelle et al., 2001). In addition, sociodemographic characteristics have been associated with significantly increased risk of mortality, in particular among females (Gillberg et al., 2010; Hirvikoski et al., 2016; Shavelle et al., 2001).

In Aotearoa New Zealand (henceforth Aotearoa NZ), large population-level research data provides a unique opportunity to examine mortality risk among Autistic children and young people (Milne et al., 2019). These data have been used previously for autism research (Bowden, Gibb, et al., 2022; McLay et al., 2021, 2022; Mujoo et al., 2023; Ruhe et al., 2022). Using an Aotearoa NZ sample may be especially important for Autistic Māori (the indigenous population of Aotearoa NZ). Māori experience a range of health-related inequities, including higher mortality rates, particularly among children and young people (McDonald et al., 2021; Mills et al., 2012; Waitangi Tribunal, 2019). Therefore, we sought to address the following two primary research questions:

1. What is the mortality risk for Autistic children and young people compared to the non-Autistic population in Aotearoa NZ?
2. Does this risk vary by sex?

And this secondary research question:

1. Within the Autistic population, are other factors such as Māori ethnicity and co-occurring intellectual disability associated with increased mortality risk?

## Methods

### Study design and participants

This is a national 15-year birth cohort study (all children born in Aotearoa NZ between the years of 1996 and 2010) using population-level data from the Integrated Data Infrastructure (IDI). The IDI contains nationwide administrative and survey data on people and households linked at the individual level (Milne et al., 2019). Managed by Stats NZ, de-identified data for research projects deemed to be beneficial to New Zealanders can only be accessed by approved researchers. The study period was chosen to balance maximising the years of follow-up time with the availability of reliable data, particularly with respect to autism diagnosis information. Mortality risk among the cohort was examined up until each individual’s 25th birthday to reflect the World Health Organization’s definition of youth.

### Mortality

The outcome of interest was all-cause mortality measured until the end of the 2021 calendar year; the latest date for complete death records. Mortality information was sourced from a combination of the Ministry of Health Mortality Collection and Births, Deaths, and Marriages data from the Department of Internal Affairs.

### Autism

The Autistic population was identified using an established IDI-based case identification method (Bowden, Thabrew, Kokaua, Audas, et al., 2020). It utilises diagnostic information captured within three national health datasets: the national minimum dataset (NMDS) (publicly funded hospital admissions), the Programme for the Integration of Mental Health Data (PRIMHD) (publicly funded secondary specialist mental health service use) and Socrates (disability support services). Autism was indicated if an autism diagnosis code was identified in any of the three datasets from birth (see Table 1 in Supplemental Appendix for more details). In the absence of any autism diagnosis code being identified, all other individuals in the birth cohort were grouped into the non-Autistic (general population) group. Due to the method’s reliance on health service use and a recorded diagnosis, in addition to the absence of data from both the primary care and outpatient settings, it is expected that it will undercount cases of autism (Bowden, Thabrew, Kokaua, Audas, et al., 2020).

**Table 1.** Sociodemographic characteristics of the participant population, 1996–2010 Aotearoa/New Zealand birth cohort, by autism status.

	Participants, no. (%)	
	Non-Autistic (*n* = 846,075)	Autistic (*n* = 11,919)
Sex		
Female	415,647 (49.1)	2577 (21.6)
Male	430,428 (50.9)	9342 (78.4)
Māori ethnicity		
Yes	262,707 (31.1)	2883 (24.2)
No	583,368 (68.9)	9306 (75.8)
Deprivation quintile		
1 (least deprived)	152,577 (18.0)	2154 (18.1)
2	143,664 (17.0)	2229 (18.7)
3	143,415 (17.0)	2271 (19.1)
4	152,157 (18.0)	2349 (19.7)
5 (most deprived)	205,428 (24.3)	2403 (20.2)
Missing	48,834 (5.8)	513 (4.3)
Urban/rural		
Urban	693,507 (82.0)	10,218 (85.7)
Rural	104,511 (12.4)	1200 (10.1)
Missing	48,057 (5.7)	501 (4.2)
Birth year		
1996	55,443 (6.6)	516 (4.3)
1997	55,281 (6.5)	549 (4.6)
1998	54,435 (6.4)	567 (4.8)
1999	54,711 (6.5)	663 (5.6)
2000	55,617 (6.6)	687 (5.8)
2001	54,201 (6.4)	714 (6.0)
2002	53,208 (6.3)	792 (6.6)
2003	54,096 (6.4)	828 (6.9)
2004	55,128 (6.5)	804 (6.7)
2005	55,410 (6.5)	873 (7.3)
2006	56,877 (6.7)	957 (8.0)
2007	60,255 (7.1)	1017 (8.5)
2008	60,702 (7.2)	927 (7.8)
2009	60,333 (7.1)	981 (8.2)
2010	60,384 (7.1)	1044 (8.8)

### Intellectual disability

The method for identifying co-occurring intellectual disability was similar to that of autism identification and also based on an established method (Bowden, Thabrew, Kokaua, Audas, et al., 2020). Among the Autistic population, diagnostic information sourced from PRIMHD, NMDS and Socrates was used, and intellectual disability indicated if an intellectual disability code was identified in any of those datasets (see Table 2 in Supplemental Appendix for more details).

**Table 2.** Observed mortality rates, by autism status.

	Participants, no. (%)	Participants, no. (%)
	Non-Autistic (*n* = 846,075)	Autistic (*n* = 11,919)
Overall	1992 (0.24)	66 (0.55)
Pre-COVID period	1434 (0.17)	48 (0.40)
COVID period	558 (0.07)	18 (0.15)
Sex		
Male	1239 (0.29)	42 (0.45)
Female	753 (0.18)	21 (0.81)
Co-occurring intellectual disability		
Yes	n/a	30 (0.89)
No	n/a	36 (0.42)

n/a: not applicable.

### Sociodemographic variables

Sex (male/female) and Māori ethnicity were sourced from the IDI personal details table. We were restricted to using existing official statistical standards for sex for the study period, which were female/male. Changes to the statistical standard for sex and gender identity that address issues including limited inclusiveness of intersex and transgender populations are now being implemented based on Stats NZ consultation undertaken in 2021 (Statistics New Zealand, 2021). Area-level deprivation and urban/rural classification of residence were determined using residence data extracted from the address notification table and measured at birth. Deprivation was measured using the New Zealand Index of Deprivation, 2013 (NZDep) (Atkinson et al., 2014). NZDep is an area-level measure of deprivation (approximately 100–200 residents) that combines Census information on a range of domains, including employment, income, education level and home ownership, to create a single continuous index. For the present study, the index was collapsed into quintiles, with one representing the least deprived households and five representing the highest level of deprivation. Using the Stats NZ urban/rural classification, five categories of rurality were established: (1) main urban (population of at least 30,000), (2) secondary urban (population 10,000–29,999), (3) minor urban (population 1000–9999), (4) rural centre (population 300–999) and (5) other rural (population < 300). These were collapsed into two groups to create a binary indicator: urban (main urban, secondary urban and minor urban area) and rural (rural centre and other rural). Age (time varying) was determined annually as at 31 December using date of birth and grouped in 5-year intervals (0–4, 5–9, 10–14, 15–19 and 20–24 years).

### Statistical analysis

Risk (hazard) of mortality was determined using Cox proportional hazard (time-to-event) regression models with a time-varying binary step function to control for the impact of the COVID-19 pandemic where ‘0’ indicated the pre-pandemic period and ‘1’ indicated the COVID period. The start of the COVID period was defined as being from the date of the first registered COVID case in New Zealand, 28 February 2020. The unit of time measurement was set to months to ensure a dataset of manageable size for analysis.

For the Autistic population, the start date for the mortality observation event window was set to the date of the first occurrence of a health service interaction with an autism diagnosis. This was because the autism case identification method requires health service utilisation which necessitates that the individual is alive. Frequency matching on age (in years) at first health service interaction, by birth year was used to artificially impose a start date for the event window for the non-Autistic population. This was undertaken to ensure that the distribution of age at the start of the event window was the same for both the Autistic and non-Autistic populations. Participants from the non-Autistic population were excluded if their imposed start date occurred after death or after permanent emigration from Aotearoa NZ (using customs data). Participants were right censored if they travelled overseas and did not return to Aotearoa NZ or were still alive at the end of the study period (i.e. 31 December 2021).

Unadjusted and adjusted hazard ratios (HRs) were generated at a population level for the association between autism and mortality to determine mortality risk for Autistic young people compared to the non-Autistic population (research question 1). The adjusted model included sex, age, Māori ethnicity, deprivation, urban/rural classification of residence, year of birth and the COVID step function. Two-tailed α = 0.05 were used to determine significance. This model was then stratified by sex (male/female) to determine the mortality risk for Autistic young people compared to the non-Autistic population for males and females, respectively (research question 2). Finally, a regression model was estimated within the Autistic population to determine the impact of co-occurring intellectual disability on mortality and other demographic factors such as ethnicity and deprivation (research question 3). The model regressed mortality on intellectual disability, sex, age, Māori ethnicity, deprivation, urban/rural classification of residence, year of birth and the COVID step function.

### Community involvement

Our research recognises the significance of engaging with the Autistic (i.e. Autistic people) and autism (e.g. parents, caregivers, family and professionals that regularly support Autistic people) communities, fostering meaningful collaboration and co-production of knowledge. One of the co-authors in this study holds the unique perspective of being an Autistic adult who is not only a mother to an Autistic adult son but also a grandmother to an Autistic child. She represents both the Autistic and autism communities in a range of advocacy roles. Three further co-authors are parents or family members of Autistic children. The authorship team also includes two clinicians who each work closely with Autistic children, their families and their communities. Each of these co-authors contributed to the study design, interpretation of results and drafting of the article.

## Results

There were 895,707 individuals in the 1996–2010 birth cohort. Of those 5946 (0.7%) were excluded because they died before the follow-up start date, and 31,767 (3.5%) were excluded because they emigrated from Aotearoa NZ before the follow-up start date. This yielded an analytical sample of 857,994 young people of whom 11,919 (1.4%) were identified as Autistic.

The sociodemographic characteristics of the analytical sample are presented in Table 1. The Autistic group was more likely to be male, and less likely to identify as Māori, be from areas of high deprivation, or live in rural areas. The non-Autistic cohort was almost uniformly distributed by birth year, while the Autistic cohort increased by birth year (see Tables 3 and 4 in Supplemental Appendix for the sociodemographic characteristics of the analytical sample, stratified by sex). Among the Autistic cohort, 3369 (29.6%) were identified with a co-occurring intellectual disability (see Table 5 in Supplemental Appendix for the sociodemographic characteristics of the Autistic sample by co-occurring intellectual disability status). Due to frequency matching on age, the age at start date was similarly distributed for both the Autistic and non-Autistic groups, both with median age at start date of 7 years (lower quartile = 4 years, upper quartile = 11 years; see Table 6 in Supplemental Appendix for more details).

**Table 3.** Unadjusted and adjusted hazard ratios (HRs) and associational 95% confidence intervals (CIs) for Cox proportional hazard regression of mortality on autism and sociodemographic characteristics with time-varying COVID step function (*N* = 93,266,640).

	Unadjusted	Adjusted
	HR (95% CI)	HR (95% CI)
Autism		
Yes	2.36 (1.83, 3.05)	2.35 (1.80, 3.06)
No	reference	
Sex		
Female	0.63 (0.58, 0.69)	0.64 (0.58, 0.70)
Male	reference	
Age (years)		
0–4	0.30 (0.21, 0.42)	0.35 (0.24, 0.51)
5–9	0.16 (0.13, 0.20)	0.19 (0.15, 0.24)
10–14	0.21 (0.18, 0.24)	0.25 (0.20, 0.30)
15–19	0.67 (0.60, 0.75)	0.75 (0.66, 0.87)
20–24	reference	
Māori ethnicity		
Yes	1.86 (1.71, 2.03)	1.61 (1.47, 1.78)
No	reference	
Deprivation quintile		
1 (least deprived)	reference	
2	1.26 (1.06, 1.50)	1.19 (1.00, 1.42)
3	1.35 (1.14, 1.60)	1.25 (1.05, 1.49)
4	1.76 (1.50, 2.07)	1.62 (1.37, 1.91)
5 (most deprived)	2.07 (1.78, 2.41)	1.82 (1.55, 2.13)
Residential location		
Urban	reference	
Rural	1.39 (1.23, 1.57)	1.50 (1.33, 1.70)
Birth year		
1996	reference	
1997	0.90 (0.77, 1.05)	0.92 (0.78, 1.09)
1998	0.86 (0.73, 1.01)	0.95 (0.80, 1.13)
1999	0.74 (0.62, 0.88)	0.80 (0.66, 0.96)
2000	0.66 (0.55, 0.79)	0.76 (0.63, 0.93)
2001	0.62 (0.50, 0.75)	0.73 (0.59, 0.90)
2002	0.60 (0.49, 0.74)	0.77 (0.62, 0.97)
2003	0.63 (0.51, 0.79)	0.80 (0.63, 1.02)
2004	0.46 (0.36, 0.59)	0.64 (0.49, 0.84)
2005	0.46 (0.34, 0.57)	0.67 (0.50, 0.89)
2006	0.47 (0.36, 0.61)	0.77 (0.57, 1.03)
2007	0.37 (0.27, 0.50)	0.77 (0.55, 1.07)
2008	0.34 (0.25, 0.47)	0.69 (0.48, 0.98)
2009	0.36 (0.26, 0.51)	0.75 (0.52, 1.08)
2010	0.39 (0.28, 0.56)	0.81 (0.55, 1.18)
COVID step function		
Yes	1.30 (1.17, 1.45)	1.13 (0.99, 1.24)
No	reference	

**Table 4.** Unadjusted and adjusted hazard ratios (HRs) and associational 95% confidence intervals (CIs) for Cox proportional hazard regression of mortality on autism and sociodemographic characteristics with time-varying COVID step function among males (*N* = 47,737,194).

	Unadjusted	Adjusted
	HR (95% CI)	HR (95% CI)
Autism		
Yes	1.54 (1.12, 2.12)	1.82 (1.32, 2.52)
No	reference	
Age (years)		
0–4	0.28 (0.19, 0.43)	0.36 (0.23, 0.59)
5–9	0.13 (0.10, 0.17)	0.17 (0.13, 0.24)
10–14	0.17 (0.14, 0.21)	0.22 (0.17, 0.28)
15–19	0.63 (0.55, 0.73)	0.73 (0.61, 0.86)
20–24	reference	
Māori ethnicity		
Yes	1.70 (1.52, 1.90)	1.52 (1.35, 1.72)
No	reference	
Deprivation quintile		
1 (least deprived)	reference	
2	1.34 (1.08, 1.67)	1.26 (1.02, 1.57)
3	1.33 (1.07, 1.65)	1.24 (1.00, 1.54)
4	1.79 (1.46, 2.19)	1.68 (1.37, 2.06)
5 (most deprived)	1.93 (1.59, 2.34)	1.75 (1.43, 2.14)
Residential location		
Urban	reference	
Rural	1.54 (1.32, 1.78)	1.63 (1.40, 1.90)
Birth year		
1996	reference	
1997	0.93 (0.77, 1.13)	0.95 (0.77, 1.16)
1998	0.83 (0.67, 1.01)	0.92 (0.74, 1.13)
1999	0.65 (0.52, 0.81)	0.69 (0.55, 0.88)
2000	0.64 (0.51, 0.80)	0.74 (0.58, 0.94)
2001	0.64 (0.50, 0.81)	0.79 (0.61, 1.03)
2002	0.56 (0.43, 0.73)	0.72 (0.54, 0.97)
2003	0.59 (0.45, 0.77)	0.79 (0.58, 1.06)
2004	0.38 (0.27, 0.52)	0.54 (0.37, 0.77)
2005	0.46 (0.33, 0.63)	0.72 (0.51, 1.03)
2006	0.36 (0.25, 0.53)	0.60 (0.40, 0.91)
2007	0.28 (0.18, 0.43)	0.59 (0.37, 0.94)
2008	0.26 (0.16, 0.41)	0.52 (0.31, 0.86)
2009	0.33 (0.22, 0.51)	0.72 (0.45, 1.16)
2010	0.33 (0.20, 0.52)	0.70 (0.42, 1.16)
COVID step function		
Yes	1.26 (1.10, 1.44)	1.10 (0.93, 1.29)
No	reference	

**Table 5.** Unadjusted and adjusted hazard ratios (HRs) and associational 95% confidence intervals (CIs) for Cox proportional hazard regression of mortality on autism and sociodemographic characteristics with time-varying COVID step function among females (*N* = 45,529,446).

	Unadjusted	Adjusted
	HR (95% CI)	HR (95% CI)
Autism		
Yes	5.58 (3.65, 8.52)	5.40 (3.42, 8.52)
No	reference	
Age (years)		
0–4	0.33 (0.19, 0.56)	0.34 (0.19, 0.63)
5–9	0.22 (0.16, 0.30)	0.22 (0.15, 0.33)
10–14	0.28 (0.22, 0.36)	0.30 (0.22, 0.41)
15–19	0.74 (0.61, 0.91)	0.81 (0.65, 1.02)
20–24	reference	
Māori ethnicity		
Yes	2.16 (1.87, 2.49)	1.78 (1.52, 2.08)
No	reference	
Deprivation quintile		
1 (least deprived)	reference	
2	1.13 (0.84, 1.52)	1.07 (0.80, 1.45)
3	1.39 (1.05, 1.85)	1.28 (0.96, 1.70)
4	1.71 (1.30, 2.23)	1.52 (1.15, 1.99)
5 (most deprived)	2.32 (1.82, 2.97)	1.93 (1.49, 2.49)
Residential location		
Urban	reference	
Rural	1.17 (0.95, 1.44)	1.29 (1.04, 1.60)
Birth year		
1996	reference	
1997	0.84 (0.63, 1.10)	0.86 (0.64, 1.16)
1998	0.91 (0.69, 1.20)	1.02 (0.76, 1.36)
1999	0.91 (0.69, 1.20)	0.99 (0.74, 1.34)
2000	0.70 (0.51, 0.95)	0.80 (0.58, 1.12)
2001	0.58 (0.41, 0.81)	0.62 (0.43, 0.91)
2002	0.68 (0.48, 0.95)	0.86 (0.60, 1.25)
2003	0.72 (0.51, 1.02)	0.84 (0.57, 1.24)
2004	0.61 (0.42, 0.89)	0.83 (0.55, 1.25)
2005	0.41 (0.26, 0.65)	0.58 (0.36, 0.96)
2006	0.66 (0.45, 0.98)	1.05 (0.67, 1.63)
2007	0.54 (0.34, 0.83)	1.06 (0.65, 1.74)
2008	0.49 (0.31, 0.79)	0.97 (0.58, 1.63)
2009	0.42 (0.25, 0.71)	0.80 (0.45, 1.43)
2010	0.52 (0.31, 0.87)	0.99 (0.56, 1.75)
COVID step function		
Yes	1.39 (1.16, 1.65)	1.19 (0.97, 1.47)
No	reference	

**Table 6.** Unadjusted and adjusted hazard ratios (HRs) and associational 95% confidence intervals (CIs) for Cox proportional hazard regression of mortality on intellectual disability and sociodemographic characteristics with time-varying COVID step function among Autistic children and young people (*N* = 1,262,382).

	Unadjusted	Adjusted
	HR (95% CI)	HR (95% CI)
Intellectual disability		
Yes	1.81 (1.09, 2.99)	2.02 (1.17, 3.46)
No	reference	
Sex		
Female	2.25 (1.34, 3.80)	1.78 (1.02, 3.10)
Male	reference	
Age (years)		
0–4	0.09 (0.01, 0.77)	0.08 (0.01, 0.81)
5–9	0.20 (0.08, 0.49)	0.21 (0.06, 0.64)
10–14	0.21 (0.09, 0.49)	0.24 (0.09, 0.68)
15–19	0.40 (0.19, 0.83)	0.37 (0.16, 0.88)
20–24	reference	
Māori ethnicity		
Yes	1.70 (0.99, 2.9)	1.57 (0.88, 2.79)
No	reference	
Deprivation quintile		
1 (least deprived)	reference	
2	1.30 (0.52, 3.23)	1.27 (0.51, 3.17)
3	1.06 (0.41, 2.76)	1.02 (0.39, 2.65)
4	1.47 (0.61, 3.54)	1.28 (0.52, 3.11)
5 (most deprived)	1.80 (0.77, 4.20)	1.47 (0.61, 3.53)
Residential location		
Urban	reference	
Rural	0.93 (0.37, 2.32)	0.97 (0.38, 2.46)
Birth year		
1996	reference	
1997	1.83 (0.69, 4.87)	2.27 (0.79, 6.55)
1998	0.93 (0.30, 2.88)	1.08 (0.31, 3.76)
1999	0.82 (0.26, 2.55)	1.00 (0.28, 3.51)
2000	0.88 (0.28, 2.73)	1.29 (0.38, 4.39)
2001	0.31 (0.06, 1.56)	0.53 (0.10, 2.86)
2002	1.06 (0.36, 3.19)	2.06 (0.59, 7.15)
2003	0.67 (0.19, 2.41)	1.21 (0.30, 4.94)
2004	0.18 (0.02, 1.55)	0.33 (0.04, 3.00)
2005	0.36 (0.07, 1.86)	0.70 (0.12, 3.94)
2006	0.19 (0.02, 1.59)	0.35 (0.04, 3.29)
2007	0.19 (0.02, 1.61)	0.37 (0.04, 3.50)
2008	0.66 (0.16, 2.73)	1.30 (0.27, 6.27)
2009	0.66 (0.16, 2.71)	1.25 (0.26, 6.10)
2010	0.23 (0.03, 1.94)	0.43 (0.03, 4.14)
COVID step function		
Yes	0.97 (0.50, 1.87)	0.83 (0.39, 1.80)
No	reference	

Table 2 displays overall observed mortality rates (from the observation start date until the end of 2021), as well as observed pre- and post-COVID mortality rates, by autism status and stratified by sex. Overall, observed mortality rates were over twice as high among Autistic compared to non-Autistic young people. This increased rate of mortality was observed in both the pre- and post-COVID periods. Mortality rates were almost twice as high for Autistic males compared to non-Autistic males, but over four times as high for Autistic females compared to non-Autistic females. Table 2 also shows that among Autistic children and young people, the observed mortality rate for those with a co-occurring intellectual disability was just over twice as high as those without a co-occurring intellectual disability.

Unadjusted and adjusted HRs representing the mortality risk for autism at a population level are presented in Table 3 (research question 1). The analysis encompassed 8,319,117 person-years of follow-up time (110,395 person-years among the Autistic group, and 8,208,722 for the non-Autistic group). Among the Autistic group, 387 (3.2%) were right censored due to leaving Aotearoa NZ during the study period and not returning, and 10,980 (92.2%) were right censored due to follow-up time elapse. Among the non-Autistic group, these numbers were 87,096 (10.3%) and 711,522 (84.2%), respectively. In adjusted analyses, autism was associated with a significant increased risk of mortality (HR = 2.35; 95% confidence interval (CI) = 1.80–3.06). Unadjusted and adjusted HRs representing the mortality risk for autism stratified by sex are presented in Tables 4 and 5 (research question 2). Among males, after adjustment, autism was associated with a significant increased risk of mortality (HR = 1.82; 95% CI = 1.32–2.52). However, this estimated risk was significantly higher among females (HR = 5.40; 95% CI = 3.42–8.52). Unadjusted and adjusted HRs representing the mortality risk for intellectual disability and other sociodemographic factors among Autistic children and young people are presented in Table 6 (research question 3). These results show that Autistic children and young people with a co-occurring intellectual disability were at significantly higher risk of mortality compared to Autistic children and young people without a co-occurring intellectual disability (HR = 2.02; 95% CI = 1.17–3.46). In addition, Autistic Māori had an estimated HR of mortality of 1.57 (95% CI = 0.89–2.79, *p* = 0.12), which was not significantly different than non-Māori, but this may reflect relatively low statistical power.

## Discussion

This 15-year birth cohort study is one of the first to examine mortality risk among Autistic children and young people at a population level, before and during the period of the COVID-19 pandemic. The estimated mortality risk for Autistic individuals was 2.35 times that of non-Autistic individuals and substantially higher among females (HR = 5.40) than males (HR = 1.82). Moreover, among Autistic children and young people, co-occurring intellectual disability was associated with almost twice (HR = 2.02) the risk of mortality compared with Autistic individuals without intellectual disability.

Our findings are consistent with existing literature. While focused on adult samples, a recent meta-analysis found that across 12 studies, the pooled rate ratio for Autistic compared to non-Autistic individuals was 2.37 (Catalá-López et al., 2022). Likewise, the only two known studies examining mortality risk among Autistic children found HRs of 2.3; 95% CI, 2.1–2.7 (Yoo et al., 2022) and 2.5, 95% CI: 2.2–2.9 (Kim et al., 2021). Our finding that mortality risk is substantially higher among young females compared to males is consistent with similar child-focused studies (Kim et al., 2021; Yoo et al., 2022) and generally aligns with those based on adult samples (Catalá-López et al., 2022). Co-occurring intellectual disability has also previously been associated with increased risk of mortality among Autistic individuals (Hwang et al., 2019; Schendel et al., 2016).

There are a range of reasons why Autistic children and young people may have a higher mortality risk than their non-Autistic counterparts. A number of conditions known to be associated with reduced life expectancy commonly co-occur among Autistic young people at rates higher than the general population (American Psychiatric Association, 2013; Isaksen et al., 2013; Liu et al., 2021; Lugo-Marin et al., 2019; Maenner et al., 2023; Postorino et al., 2016; Simonoff et al., 2008; Thurman et al., 2017; Walker et al., 2015). This includes epilepsy, intellectual disability, and in particular, the presence of mental health (e.g. anxiety, depression and schizophrenia) or behavioural (e.g. attention-deficit hyperactivity disorder (ADHD), conduct disorder and oppositional defiant disorder) conditions, all of which have been associated with an elevated risk of mortality among Autistics (Hwang et al., 2019; Mouridsen et al., 2008; Pickett et al., 2011; Schendel et al., 2016). Moreover, sequelae associated with co-occurring mental health and neurodevelopmental conditions among Autistic young people include increased risk of intentional (e.g. self-harm) and unintentional injury (e.g. suffocation, drowning), and harm from others (e.g. caregiver maltreatment) that can result in death (Frederick et al., 2019; Guan et al., 2022; Hirvikoski et al., 2016; Schendel et al., 2016; Shavelle et al., 2001).

While several studies have determined that the mortality risk among Autistic females is higher compared to Autistic males, our understanding of why is limited. The predominant hypothesis is that females diagnosed with autism are more likely to present with complex needs including higher rates of co-occurring neurological and neurodevelopmental conditions (Gillberg et al., 2010; Hwang et al., 2019). Therefore, the different clinical characteristics of these groups may underlie this finding.

The findings should also be viewed in the context of the social model of disability, a framework that seeks to understand disability as a social construct rather than a purely medical or individual issue (Oliver, 2018). From this perspective, disability is viewed as a process caused by societal barriers, attitudes and exclusionary practices. In regard to the current study, any interpretation of findings must acknowledge the importance of societal responsibility with an emphasis on system change in areas such as health, disability support and education to better support the needs and well-being of Autistic children and their families.

Indeed, our findings indicate that Autistic people and their families require better supports and improved access to effective health care. Known differences in social communication, cognition and learning mean that there are many barriers to accessing timely diagnosis for their health concerns and prompt care (Malik-Soni et al., 2022; Smith et al., 2020). Moreover, an under-resourced health system coupled with a substantial recent increase in autism prevalence means that there are significant waitlists for specific autism-related supports. In combination, this presents complex challenges for the health system to provide, and for Autistic young people and their families to access, timely, appropriate and equitable care.

Increased clinical awareness and understanding of autism, including common co-occurring conditions and their associated risks, is required to build the capacity and ability to care for this population. Moreover, programmes to enable Autistic people to communicate their symptoms and seek help when needed would help decrease the difficulties in getting appropriate access to health care and social support. In addition, studies have shown a link between psychological vulnerabilities due to social and communication differences and high rates of depression, anxiety and suicidal ideation (Buck et al., 2014). Therefore, tailoring mental health support for Autistic young people may help to prevent escalation of these issues. In addition, Autistic young people often come from socio-economically disadvantaged environments and are more likely to be exposed to environmental risk factors such as substance misuse and violence (Roman-Urrestarazu et al., 2021). Specific supports and programmes targeted towards the creation of safer living environments are required. Overall efforts to improve access to healthcare and the determinants of health would improve outcomes for Autistic people.

The finding of a non-significant HR of 1.57 for Autistic Māori compared to Autistic non-Māori is important for this population. While caution is needed in interpreting this finding, given the lack of statistical significance which is due to the small number of Autistic Māori and the rarity of the mortality outcome, the association of increased risk of mortality for Autistic Māori is consistent with the increased mortality risk for Māori in the general population (McDonald et al., 2021). While our understanding of the reasons for this are limited, it may be related to the increased risk of avoidable mortality for young Māori in the general population (Mills et al., 2012; Peden et al., 2021; Suicide Mortality Review Committee, 2016; Young et al., 2021). Further research is needed; however, this finding supports the continuation of this analysis with an expanded cohort as more data becomes available.

There are many strengths in this study. The use of linked population-level administrative data held within the IDI enabled construction of a large contemporary national birth cohort, identification of those with an autism diagnosis and the ability to control for a range of important sociodemographic measures. Contemporary data also enabled analysis during the COVID-19 pandemic period. However, the study must also be viewed in the context of several limitations. While the study utilised a large 15-year birth cohort, the rarity of mortality among this population and the corresponding small numbers within the Autistic population meant that analyses of cause of death or age at death were not possible. In addition, the method for identifying autism diagnoses in the data, while used frequently in existing literature (Bowden, 2023; Bowden, Milne, et al., 2022; Bowden, Thabrew, Kokaua, & Braund, 2020; McLay et al., 2021, 2022; Mujoo et al., 2023; Ruhe et al., 2022), remains unvalidated and likely undercounts true cases of autism (Bowden, Thabrew, Kokaua, Audas, et al., 2020; Ruhe et al., 2022). In addition, the method is restricted to diagnosis information on autism that is captured within the health setting only. Other settings, such as education, do not routinely collect and record autism diagnosis information in Aotearoa NZ.

The approach developed for this study could be reapplied as more data become available and sample sizes can be increased, enabling investigation of specific causes of death among this population, age at death and any relationship with co-occurring conditions. An alternative might be to create a bespoke research dataset linking health data to data contained within the Mortality Review Database which may enable a sample size large enough for analysis (Child and Youth Mortality Review Committee, 2002). Likewise, future research could examine predictors of increased risk of mortality among the Autistic population, including common co-occurring neurodevelopmental and mental health conditions as well as key sociodemographic characteristics in order to inform preventive interventions.

## Conclusion

In this nationwide birth cohort study, autism was associated with higher mortality in youth compared to the non-Autistic population. Mortality risk was particularly high for Autistic females and those with co-occurring intellectual disability. Increased efforts are required to better meet the health needs of this population.

## Supplemental Material

sj-docx-1-aut-10.1177_13623613231224015 – Supplemental material for Mortality risk among Autistic children and young people: A nationwide birth cohort study Supplemental material, sj-docx-1-aut-10.1177_13623613231224015 for Mortality risk among Autistic children and young people: A nationwide birth cohort study by Hien Vu, Nicholas Bowden, Sheree Gibb, Richard Audas, Joanne Dacombe, Laurie McLay, Andrew Sporle, Hilary Stace, Barry Taylor, Hiran Thabrew, Reremoana Theodore, Jessica Tupou and Philip J Schluter in Autism
