# Supplementary material for: Mortality risk among Autistic children and young people: A nationwide birth cohort study
Source: Autism. 2024 Feb 4;28(9):2244–53. doi: 10.1177/13623613231224015 (PMC11395172; doi:10.1177/13623613231224015)
Supplement: sj-docx-1-aut-10.1177_13623613231224015 – Supplemental material for Mortality risk among Autistic children and young people: A nationwide birth cohort study [file sj-docx-1-aut-10.1177_13623613231224015.docx]

## Supplementary Files

eTable 1: Diagnostic codes for identifying autism

| **Dataset** | **Code Type** | **Code** | **Code Description** |
| --- | --- | --- | --- |
| NMDS & PRIMHD | ICD-10-AM | F84.0 | Autistic disorder |
| NMDS & PRIMHD | ICD-10-AM | F84.1 | Atypical autism |
| NMDS & PRIMHD | ICD-10-AM | F84.3 | Other childhood disintegrative disorder |
| NMDS & PRIMHD | ICD-10-AM | F84.5 | Asperger’s syndrome |
| NMDS & PRIMHD | ICD-10-AM | F84.8 | Other pervasive developmental disorders |
| NMDS & PRIMHD | ICD-10-AM | F84.9 | Pervasive developmental disorder, unspecified |
| PRIMHD | DSM-IV | 299.00 | Autistic disorder |
| PRIMHD | DSM-IV | 299.10 | Other childhood disintegrative disorder |
| PRIMHD | DSM-IV | 299.80 | Asperger’s disorder/pervasive development disorder NOS |
| Socrates | Assigned Diagnosis | 1206 | Asperger’s syndrome |
| Socrates | Assigned Diagnosis | 1207 | Other ASD |
| Socrates | Assigned Diagnosis | 1211 | Autism spectrum disorder |

NMDS – National Minimum Dataset

PRIMHD – Programme for the Integration of Mental Health Data

ICD-10-AM - International Statistical Classification of Diseases and Related Health Problems, Tenth Revision, Australian Modification

DSM-IV - Diagnostic and Statistical Manual of Mental Disorders, 4th edition

eTable 2: Diagnostic codes for identifying intellectual disability

| **Dataset** | **Code Type** | **Code** | **Code Description** |
| --- | --- | --- | --- |
| NMDS & PRIMHD | ICD-10-AM | F70.x | Mild mental retardation |
| NMDS & PRIMHD | ICD-10-AM | F71.x | Moderate mental retardation |
| NMDS & PRIMHD | ICD-10-AM | F72.x | Severe mental retardation |
| NMDS & PRIMHD | ICD-10-AM | F73.x | Profound mental retardation |
| NMDS & PRIMHD | ICD-10-AM | F78.x | Other mental retardation |
| NMDS & PRIMHD | ICD-10-AM | F79.x | Unspecified mental retardation |
| PRIMHD | DSM-IV | 317.x | Mild mental retardation |
| PRIMHD | DSM-IV | 318.0 | Moderate mental retardation |
| PRIMHD | DSM-IV | 318.1 | Severe mental retardation |
| PRIMHD | DSM-IV | 318.2 | Profound mental retardation |
| PRIMHD | DSM-IV | 319.x | mental retardation, severity unspecified |
| PRIMHD | Team Type | 12 | Intellectual Disability Dual Diagnosis Team |
| Socrates | Assigned Diagnosis | 1208 | intellectual disability, type not specified |
| Socrates | Assigned Diagnosis | 1209 | learning disability, type not specified |
| Socrates | Assigned Diagnosis | 1210 | developmental delay, type not specified |
| Socrates | Assigned Diagnosis | 1299 | other intellectual, learning or developmental disorder |

NMDS – National Minimum Dataset

PRIMHD – Programme for the Integration of Mental Health Data

ICD-10-AM - International Statistical Classification of Diseases and Related Health Problems, Tenth Revision, Australian Modification

DSM-IV - Diagnostic and Statistical Manual of Mental Disorders, 4th edition

Team Type – A code which described the team who provided a service

eTable 3: Sociodemographic characteristics of the participant female population, 1996-2010 Aotearoa/New Zealand birth cohort, by autism status.

|  | **Participants, No. (%)** | |
| --- | --- | --- |
|  | **Non-autistic (n=415,647)** | **Autistic (n=2,577)** |
| *Ethnicity* |  |  |
| EO | 304,392 (73.2) | 2,229 (86.5) |
| Māori | 128,571 (30.9) | 621 (24.1) |
| Pasifika | 62,958 (15.1) | 177 (6.9) |
| Asian | 38,343 (9.2) | 201 (7.8) |
| MELAA | 5,469 (1.3) | 39 (1.5) |
| *Deprivation quintile* |  |  |
| 1 (least deprived) | 751,14 (18.1) | 462 (17.9) |
| 2 | 70,773 (17.0) | 498 (19.3) |
| 3 | 70,683 (17.0) | 477 (18.5) |
| 4 | 74,853 (18.0) | 522 (20.3) |
| 5 (most deprived) | 100,617 (24.2) | 513 (19.9) |
| *Urban/Rural* |  |  |
| Urban | 341,271 (82.1) | 2217 (86.0) |
| Rural | 51,138 (12.3) | 258 (10.0) |
| *Birth Year* |  |  |
| 1996 | 27,045 (6.5) | 111 (4.3) |
| 1997 | 27,165 (6.5) | 129 (5.0) |
| 1998 | 26,637 (6.4) | 120 (4.7) |
| 1999 | 26,871 (6.5) | 156 (6.1) |
| 2000 | 27,267 (6.6) | 153 (5.9) |
| 2001 | 26,901 (6.5) | 156 (6.1) |
| 2002 | 26,226 (6.3) | 183 (7.1) |
| 2003 | 26,574 (6.4) | 168 (6.5) |
| 2004 | 27,090 (6.5) | 198 (7.7) |
| 2005 | 27,207 (6.5) | 198 (7.7) |
| 2006 | 28,071 (6.8) | 207 (8.0) |
| 2007 | 29,631 (7.1) | 204 (7.9) |
| 2008 | 29,730 (7.2) | 171 (6.6) |
| 2009 | 29,580 (7.1) | 198 (7.7) |
| 2010 | 29,655 (7.1) | 228 (8.8) |

eTable 4: Sociodemographic characteristics of the participant male population, 1996-2010 Aotearoa/New Zealand birth cohort, by autism status.

|  | **Participants, No. (%)** | |
| --- | --- | --- |
|  | **Non-autistic (n=430,428)** | **Autistic (n=9,342)** |
| *Ethnicity* |  |  |
| EO | 313,335 (72.8) | 7,917 (84.7) |
| Māori | 134,136 (31.2) | 2,265 (24.2) |
| Pasifika | 65,184 (15.1) | 762 (8.2) |
| Asian | 39,609 (9.2) | 870 (9.3) |
| MELAA | 5952 (1.4) | 114 (1.2) |
| *Deprivation quintile* |  |  |
| 1 (least deprived) | 77,463 (18.0) | 1,695 (18.1) |
| 2 | 72,888 (16.9) | 1,725 (18.5) |
| 3 | 72,732 (16.9) | 1,791 (19.2) |
| 4 | 77,304 (18.0) | 1,830 (19.6) |
| 5 (most deprived) | 104,808 (24.3) | 1,893 (20.3) |
| *Urban/Rural* |  |  |
| Urban | 352236 (81.8) | 7,998 (85.6) |
| Rural | 53376 (12.4) | 942 (10.1) |
| *Birth Year* |  |  |
| 1996 | 28,395 (6.6) | 405 (4.3) |
| 1997 | 28,119 (6.5) | 420 (4.5) |
| 1998 | 27,795 (6.5) | 447 (4.8) |
| 1999 | 27,840 (6.5) | 504 (5.4) |
| 2000 | 28,350 (6.6) | 534 (5.7) |
| 2001 | 27,300 (6.3) | 561 (6.0) |
| 2002 | 26,979 (6.3) | 612 (6.6) |
| 2003 | 27,522 (6.4) | 660 (7.1) |
| 2004 | 28,038 (6.5) | 606 (6.5) |
| 2005 | 28,203 (6.6) | 678 (7.3) |
| 2006 | 28,803 (6.7) | 753 (8.1) |
| 2007 | 30,624 (7.1) | 816 (8.7) |
| 2008 | 30,972 (7.2) | 759 (8.1) |
| 2009 | 30,753 (7.1) | 780 (8.3) |
| 2010 | 30,729 (7.1) | 816 (8.7) |

eTable 5: Sociodemographic characteristics of the participant autistic population, 1996-2010 Aotearoa/New Zealand birth cohort, by co-occurring intellectual disability status.

|  | **Participants, No. (%)** | |
| --- | --- | --- |
|  | **No intellectual disability (n=8,550)** | **Intellectual disability (n=3,369)** |
| *Sex* |  |  |
| Female | 1,812 (21.2) | 768 (22.8) |
| Male | 6,738 (78.8) | 2,601 (77.2) |
| *Ethnicity* |  |  |
| EO | 7,590 (88.8) | 2,550 (75.7) |
| Māori | 1,938 (22.7) | 945 (28.0) |
| Pasifika | 513 (6.0) | 432 (12.8) |
| Asian | 639 (7.5) | 432 (12.8) |
| MELAA | 105 (1.2) | 51 (1.5) |
| *Deprivation quintile* |  |  |
| 1 (least deprived) | 1,629 (19.1) | 528 (15.7) |
| 2 | 1,692 (19.8) | 534 (15.9) |
| 3 | 1,695 (19.8) | 576 (17.1) |
| 4 | 1,644 (19.2) | 705 (20.9) |
| 5 (most deprived) | 1,533 (17.9) | 870 (25.8) |
| *Urban/Rural* |  |  |
| Urban | 7,269 (85.0) | 2,943 (87.4) |
| Rural | 930 (10.9) | 273 (8.1) |
| *Birth Year* |  |  |
| 1996 | 357 (4.2) | 156 (4.6) |
| 1997 | 408 (4.8) | 141 (4.2) |
| 1998 | 411 (4.8) | 159 (4.7) |
| 1999 | 477 (5.6) | 183 (5.4) |
| 2000 | 474 (5.5) | 213 (6.3) |
| 2001 | 513 (6.0) | 201 (6.0) |
| 2002 | 573 (6.7) | 219 (6.5) |
| 2003 | 588 (6.9) | 240 (7.1) |
| 2004 | 582 (6.8) | 219 (6.5) |
| 2005 | 645 (7.5) | 231 (6.9) |
| 2006 | 681 (8.0) | 273 (8.1) |
| 2007 | 756 (8.8) | 264 (7.8) |
| 2008 | 663 (7.8) | 267 (7.9) |
| 2009 | 699 (8.2) | 282 (8.4) |
| 2010 | 723 (8.5) | 321 (9.5) |

eTable 6: Frequency distribution of age at reference start date

|  | **No autism (n=846,075)** | | | **Autism (n=11,922)** | | |
| --- | --- | --- | --- | --- | --- | --- |
| **Age (years)** | **n** | **Freq (%)** | **Cumulative freq. (%)** | **n** | **Freq (%)** | **Cumulative freq. (%)** |
| 1 | 4,704 | 0.56 | 0.56 | 66 | 0.55 | 0.55 |
| 2 | 37,194 | 4.40 | 4.95 | 519 | 4.35 | 4.91 |
| 3 | 80,745 | 9.54 | 14.50 | 1,158 | 9.71 | 14.62 |
| 4 | 97,371 | 11.51 | 26.00 | 1,395 | 11.70 | 26.32 |
| 5 | 82,479 | 9.75 | 35.75 | 1,161 | 9.74 | 36.06 |
| 6 | 68,547 | 8.10 | 43.85 | 960 | 8.05 | 44.11 |
| 7 | 62,304 | 7.36 | 51.22 | 873 | 7.32 | 51.43 |
| 8 | 61,683 | 7.29 | 58.51 | 885 | 7.42 | 58.86 |
| 9 | 56,385 | 6.66 | 65.17 | 810 | 6.79 | 65.65 |
| 10 | 54,483 | 6.44 | 71.61 | 807 | 6.77 | 72.42 |
| 11 | 44,640 | 5.28 | 76.89 | 648 | 5.44 | 77.86 |
| 12 | 40,173 | 4.75 | 81.64 | 570 | 4.78 | 82.64 |
| 13 | 32,295 | 3.82 | 85.45 | 459 | 3.85 | 86.49 |
| 14 | 29,253 | 3.46 | 88.91 | 408 | 3.42 | 89.91 |
| 15 | 28,101 | 3.32 | 92.23 | 378 | 3.17 | 93.08 |
| 16 | 20,490 | 2.42 | 94.65 | 276 | 2.32 | 95.40 |
| 17 | 14,478 | 1.71 | 96.37 | 186 | 1.56 | 96.96 |
| 18 | 10,803 | 1.28 | 97.64 | 135 | 1.13 | 98.09 |
| 19 | 6,615 | 0.78 | 98.42 | 81 | 0.68 | 98.77 |
| 20 | 4,407 | 0.52 | 98.95 | 51 | 0.43 | 99.19 |
| 21 | 3,822 | 0.45 | 99.40 | 45 | 0.38 | 99.57 |
| 22 | 2,799 | 0.33 | 99.73 | 30 | 0.25 | 99.82 |
| 23 | 1,518 | 0.18 | 99.91 | 15 | 0.13 | 99.95 |
| 24 | 786 | 0.09 | 100.00 | 6 | 0.05 | 100.00 |
